# Social-Skills and Parental Training plus Standard Treatment versus Standard Treatment for Children with ADHD – The Randomised SOSTRA Trial

**DOI:** 10.1371/journal.pone.0037280

**Published:** 2012-06-20

**Authors:** Ole Jakob Storebø, Christian Gluud, Per Winkel, Erik Simonsen

**Affiliations:** 1 Child Psychiatric Clinic, Child and Adolescent Psychiatric Department, Region Zealand, Denmark; 2 Psychiatric Research Unit, Region Zealand, Roskilde, Denmark; 3 Copenhagen Trial Unit, Center for Clinical Intervention Research, Department 3344, Rigshospitalet, Copenhagen University Hospital, Copenhagen, Denmark; University of Groningen, The Netherlands

## Abstract

**Objective:**

To investigate the effects of social-skills training and parental training programme for children with attention deficit hyperactivity disorder (ADHD).

**Methods:**

We conducted a randomized two-armed, parallel group, assessor-blinded superiority trial consisting of social-skills training plus parental training and standard treatment versus standard treatment alone. A sample size calculation showed at least 52 children should be included for the trial with follow up three and six months after randomization. The primary outcome measure was ADHD symptoms and secondary outcomes were social skills and emotional competences.

**Results 56:**

children (39 boys, 17 girls, mean age 10.4 years, SD 1.31) with ADHD were randomized, 28 to the experimental group and 27 to the control group. Mixed-model analyses with repeated measures showed that the time course (y  =  a + bt + ct^^2^^) of ADHD symptoms (p = 0.40), social skills (p = 0.80), and emotional competences (p = 0.14) were not significantly influenced by the intervention.

**Conclusions:**

Social skills training plus parental training did not show any significant benefit for children with attention deficit hyperactivity disorder when compared with standard treatment. More and larger randomized trials are needed.

**Trial Registration:**

ClinicalTrials.gov NCT00937469

## Introduction

Attention-deficit hyperactivity disorder (ADHD) affects 3% to 5% of all children [1]. The core ADHD symptoms include lack of attention, impulsiveness, and hyperactivity [2]; [3]. Many children and adolescents with ADHD also frequently suffer from lack of social competence and have language difficulties, learning problems, and difficulties in interacting with parents and teachers. The fundamental basis for the social skills problems are the attentional and cognitive difficulties, such as difficulties with problem solving, planning, mood regulation, and motivation delay. These difficulties are closely related to the condition [4]–[9]. Pharmacological treatment of children with ADHD shows beneficial effects on core symptoms in about 80% of the patients [10]. It is a question if pharmacological treatment alone has sufficient effect on the social skills problems [11]–[16]. In both the European clinical guidelines for hyperkinetic disorders and the NICE guidelines, parental training and social skills training for the school-aged children are recommended [17]; [18]. We have identified four meta-analyses on social skills training for children with ADHD. Two of them state that social skills training for children with ADHD has no effect [19]; [20], and two of them state that social skills training for children with ADHD has a significant beneficial treatment effect [21]; [22].

Recently, we conducted a Cochrane systematic review of randomized clinical trials to investigate the effect of social skills training for children with ADHD. Our review showed no statistically significant treatment effects either on social skills competences (positive value  =  better for the intervention group) (SMD 0.16; 95% CI −0.04 to 0.36; 5 trials, n  = 392 participants), on general behaviour (negative value  =  better for the intervention group) (SMD 0.00; 95% CI −0.21 to 0.21; 3 trials, n  = 358 participants), or on ADHD symptoms (negative value  =  better for the intervention group) (SMD −0.02; 95% CI −0.19 to 0.16; 6 trials, n  = 515 participants). Because of the high risk of bias (systematic errors) in all the included trials and insufficient power (few participants in the trials), these findings are inconclusive. All the included trials had no blinding of outcome assessment and more than half of the trials had high risk of bias regarding generation of allocation sequence and allocation concealment which makes the results questionable and inconclusive. Our review suggests that there is little evidence to support or refute social skills training for adolescents with ADHD. Therefore, there is a need for more trials, with low risk of bias and with a sufficient number of participants, investigating the efficacy of social skills training versus no training for both children and adolescents [23]. Therefore, we designed the social skills training attachment (SOSTRA) trial on the basis of this review and efforts were made to avoid systematic errors in its design [24]. Our hypothesis was that adding social skills training plus parental training to the standard treatment would give a statistical significant difference on the children’s ADHD symptoms, their social and emotional competences compared with standard treatment alone.

Some studies suggest a possible connection between insecure attachment patterns and ADHD, and the specific attachment style has a prognostic influence [25]–[28]. ADHD is an inherited disease, and parent’s own ADHD symptoms might also predict the outcome [29]. Therefore we also assessed possible predictive value of the child’s attachment competence and the parent’s ADHD symptoms.

## Methods

### Design

Previously we have described the design and plan for the analysis of the SOSTRA trial [24]. Briefly, children aged 8 to 12 years who had been diagnosed with ADHD and their parents were randomized to experimental and control treatment. The experimental treatment consisted of social-skills training plus parental training combined with standard treatment versus standard treatment alone. The design was a randomized, two-armed, parallel group, assessor-blinded superiority trial. The children were examined at baseline and three months and six months after randomization. In this trial we included a baseline assessment of the child’s attachment competence and the parent’s ADHD symptoms, and analysed the prognostic influence of these factors. The protocol for this trial and supporting CONSORT checklist are available as supporting information; see Checklist S1 and Protocol S1.

### Participants

The inclusion period for the trial was from August 2009 to January 2011.The children were suspected to have an attention deficit hyperactivity disorder and were referred to the Child Psychiatric Clinics in Holbaek or Roskilde. They were screened according to the following inclusion criteria: ADHD diagnosis according to the Diagnostic and Statistical Manual of Mental Disorders (DSM-IV, 1994), 8 years to 12 years at the time of the start of assessment, and parents willing to take part in the trial and giving consent for medical treatment of the child as well as to participation of the child in the trial. Exclusion criteria were: schizophrenia or all the autism diagnoses according to DSM IV, violent and criminal children, both verbal and nonverbal intelligence quotient (IQ) below 80, previously medicated for ADHD, and resistance against participating.

### Measures and Reliability

The children were screened at entry by the The Schedule for Affective Disorders and Schizophrenia for School-aged Children (K-SADS). This semi-structured interview includes algorithms from the DSM-IV in children and adolescents [30]. The K-SADS was administered by the first author who was trained to administer the K-SADS (OJS). The child was screened for autism and the parents completed the Social Communication Questionnaires (SCQ). Children with scores above 15 on two SCQ questionnaires were excluded [31]. The parents also completed the Adult Self-Report Scale (ASRS) to screen for adult ADHD symptoms [32]. The children who had not been subjected to the Wechsler Intelligence Scale for Children (Wisc-3 test) during the last three years were tested with the Wisc-3 test by psychologists from the Clinic [33]. We did not perform blinded assessment to check for reliability, but all the children were also assessed by the clinicians and there were 100 percent clinical consensus. The inter-rater reliability of the K-SADS have kappa values between 0.63–1.00 [34].

All of the children were tested using the Children Attachment Interview (CAI) [35]. This interview was scored by a certified rater who was blinded to the randomised interventions. The children’s teachers completed the Conner’s 3 and the Conner’s Comprehensive Behaviour Rating Scale (CBRS) with blinding to the randomised interventions [36]; [37].

### Outcome Measures

The outcomes measured at three months and six months after randomization included indexes from the Conners 3 and the Conners Comprehensive Behaviour Rating Scale (CBRS) rating scales. The primary outcome was: ‘hyperactivity/impulsitivity’. The secondary outcomes were: ‘social problems’; ‘peer relations’; ‘aggressive behaviour’; ‘emotional distress’; ‘executive functioning’; and ‘academic performances’ [36]; [37].

### Randomization and Blinding

The Copenhagen Trial Unit (CTU) conducted central randomization with computer generated, permuted randomization sequences in blocks of four with an allocation ratio of 1:1 stratified for sex and comorbidity. The block size was unknown to the investigators. A research secretary randomized the patient by calling the CTU providing a personal pin code, patient number, and information on the stratification variables.

The interventions given were not ‘blind’ to participants, parents, treating physicians, or personnel in the Child Psychiatric Clinic in Holbaek. However, the outcome assessors of the primary and secondary outcomes (the teachers) were kept blinded of the allocated intervention. The involved parties were also instructed not to inform the assessors of the intervention allocated. To secure integrity, the principal investigator hid all data that could be used to identify the patient’s allocation before data entry. Blinded data were then handed over to the CTU, which was in charge of data entry and statistical analyses. Standardized procedures including double data entry were assured.

### Ethical Considerations and Regulatory Approval

Participants were informed of the trial in writing and orally; written informed consent was obtained from the participant’s principal caregiver. There were no apparent ethical problems since all participants were offered medical treatment, and there were no known disadvantages of social-skills training; nevertheless, any adverse events of the intervention were reported. The trial obtained approval from the Regional Ethics Committee of Zealand (SJ-85), was registered at the Danish Data Protection Agency DO50892, and registered at www.clincal trials.gov NCT00937469.

### Treatment Groups

#### Standard treatment

The standard treatment offered to both the experimental group and the control group encompassed the normal practice regarding ADHD patients at the Child Psychiatric Clinic in Holbaek. After assessment and confirmation of the ADHD diagnosis, the family was offered medical treatment for the child following a medication protocol. The children had never previously received medical treatment for ADHD. We followed defined treatment algorithms for the medication of ADHD. The treatment started with the first choice: methylphenidate; the second choice: dexamphetamine; and atomoxetine was considered in patients where there was a suspicion of abuse of dexamphetamine or a significant anxiety component change. During the eight months following randomization, the children were not offered any supplementary treatment, such as anti-psychotics or antidepressants. All children were examined one week and again one month after the start of medical treatment; positive effects and adverse effects were evaluated. The standard treatment involved an educational parent group, where the parents met three times during the eight week trial and received general information about ADHD.

#### The experimental intervention

Social-skills training aimed to improve and maintain the individual’s social skills. The children were taught how to adjust their verbal and nonverbal behaviour in their social interaction. Social-skills training also included efforts to change the child’s cognitive assessment of the ‘social world’ [38]. The training generally focused on teaching the children to ‘read’ the subtle cues in social interaction, such as learning to wait for their turn [39]. The children in SOSTRA were offered weekly 90 minute social-skills training sessions in a total of eight weeks. Each group included two therapists trained in social-skills training before the trial. Therapists from the Langager School in Aarhus gave continuous supervision throughout the trial. Each session was video recorded, and the therapist completed forms confirming that he/she had followed the manual. These videos and forms were used to ensure that the planned material in the intervention was being sufficiently implemented. The intervention manual, which may be obtained from the corresponding author, conforms to the programme of several randomized trials [40]; [41]. Different methods of teaching the children social skills were used, all of which have proved successful in other social-skills programmes [42]. Didactic instructions were used, including work with symbols, games, creative techniques, music, story reading, and movies. Each session had a theme, such as self-worth, nonverbal communication, feelings, impulse control, aggression management, conflict resolution, and problem solving. Many of these themes are recommended as important topics in social skills training by the NICE guidelines [17]. The treatment focused on strengthening the ability of the children to control them to start a self-help process.

During the process where the children received social skills training, the parents attended parental training. The themes from the children’s groups were discussed during the parental groups. The children’s homework was also discussed. The efficacy of the intervention was assessed by studying the amount of improvement in ADHD symptoms and social skills per se, or by assessing psychological functioning on a broader aspect, including the quality of peer relationships and emotional competency.

### Data Analysis

The sample size was calculated on the basis of a type I error (α) of 5% and a type II error (β) of 20%, thus a power of 80%, and an allocation ratio of 1:1. With a minimally clinical relevant difference of 4 points between the intervention group and the control group on the Conner’s 3^rd^ Edition Rating Scale ‘hyperactivity-impulsivity’ subindex (the primary outcome) and an assumed standard deviation of 5 points on the same scale [43]; [44], a sample size of 26 participants in each group was needed. We based our minimal relevant difference in mean score of 4 on the primary outcome the ‘hyperactivity-impulsivity’ subindex on Conners’ scale on the existing literature and prior experience from our research department. The index Hyperactivity-Impulsivity on Conners’ scale consists of 18 items and may range from 0 to 54. We consider the difference in means to be of clinical relevance in this patient group and under the present settings [43]; [44]. If we are able to change the symptom severity by 4 or more in this hard to treat patient group we find this clinically relevant. The chosen SD, of 5, can be viewed upon as a tad low, however, under the present setting with the present population and specific intervention conditions, we foresaw limited variation. The estimate of the SD for the Hyperactivity-Impulsivity index on Connors’ scale is primarily influenced by the data from Horn et al. [44].

The statistical analysis of the outcomes was based on the ‘intention-to-treat’ principle and primarily conducted with adjustment for the protocol specified stratification variables (sex and presence of co-morbidity) and secondarily conducted without this adjustment [24]. The group coding was concealed for the statistician. The level of significance was 0.05.

The mixed-model repeated measures method (SAS version 9.1) was used to compare the effect of the two interventions over time on the outcome measures. The model is the following: Outcome measure  =  a⋅sex + b⋅co-morbidity + c⋅intervention-group + d⋅t + e⋅t^2^ + f⋅intervention-group⋅t + g⋅intervention-group⋅t^2^, where co-morbidity, sex and intervention-group are binary indicator variables and t is treated as a continuous variable; a through g are coefficients to be estimated during the analysis. The basic model is Outcome measure  =  a⋅sex + b⋅co-morbidity + d⋅t + e⋅t^2^, where the outcome measure is modelled as a linear function of time (t) and time squared (t^2^). The latter term is included to model a time course that may be almost linear initially and then blunted as time goes by. If sex or co-morbidity is having an impact on the outcome measure this effect is compensated for by including the terms sex and co-morbidity in the model to improve the precision.

To model a possible impact of the intervention on the mean level, the slope of the linear function (t), and the slope of t^2^ the terms intervention-group + intervention-group⋅t + intervention-group⋅t^2^, respectively, are added to the model.

A sequential hypothesis testing was used, which is appropriate for polynomial models. Since the measurements within a given patient are probably dependent, this dependency is modelled by a co-variance matrix, common to all patients. Initially, three types of covariance matrices were examined: compound symmetric, AR(1), and unstructured. Using the Akaike and the Schwartz Bayesian criteria, the best of these three covariance structures was chosen.

Prior to each analysis the six distributions of the outcome measure defined by time and intervention-group were examined to see if the assumption of normality was fulfilled (tests of kurtosis and skewness as well as Shapiro Wilks test (p<0.01) plus inspection of histograms and probability distributions). Prognostic factors measured were: assessment of the attachment between the child and the parents and an assessment of the parent’s own ADHD symptoms [32]; [35]. Of the 165 planned measurements per outcome measure, the percentage missing ranged from 1.3% to 7.2%. Two out of the 165 sets of questionnaires were missing. The rest of the missing data were due to inadequate answering of the questionnaires and resulted in a few missed indexes on some of the participants.

For the purpose of this trial the baseline values of the variables CAI group, ASRS score (father), and ASRS score (mother) were re-coded into binary variables (CAI-binary, ASRS (father)-binary, and ASRS (mother)-binary, respectively). (ASRS: 0 =  scores 1 to 3; 1 =  scores 4 to 6. CAI: 0 =  secure, insecure/preoccupied, insecure/dismissing; 1 =  disorganized/secure and disorganized/not secure).

## Results

**Figure 1:**
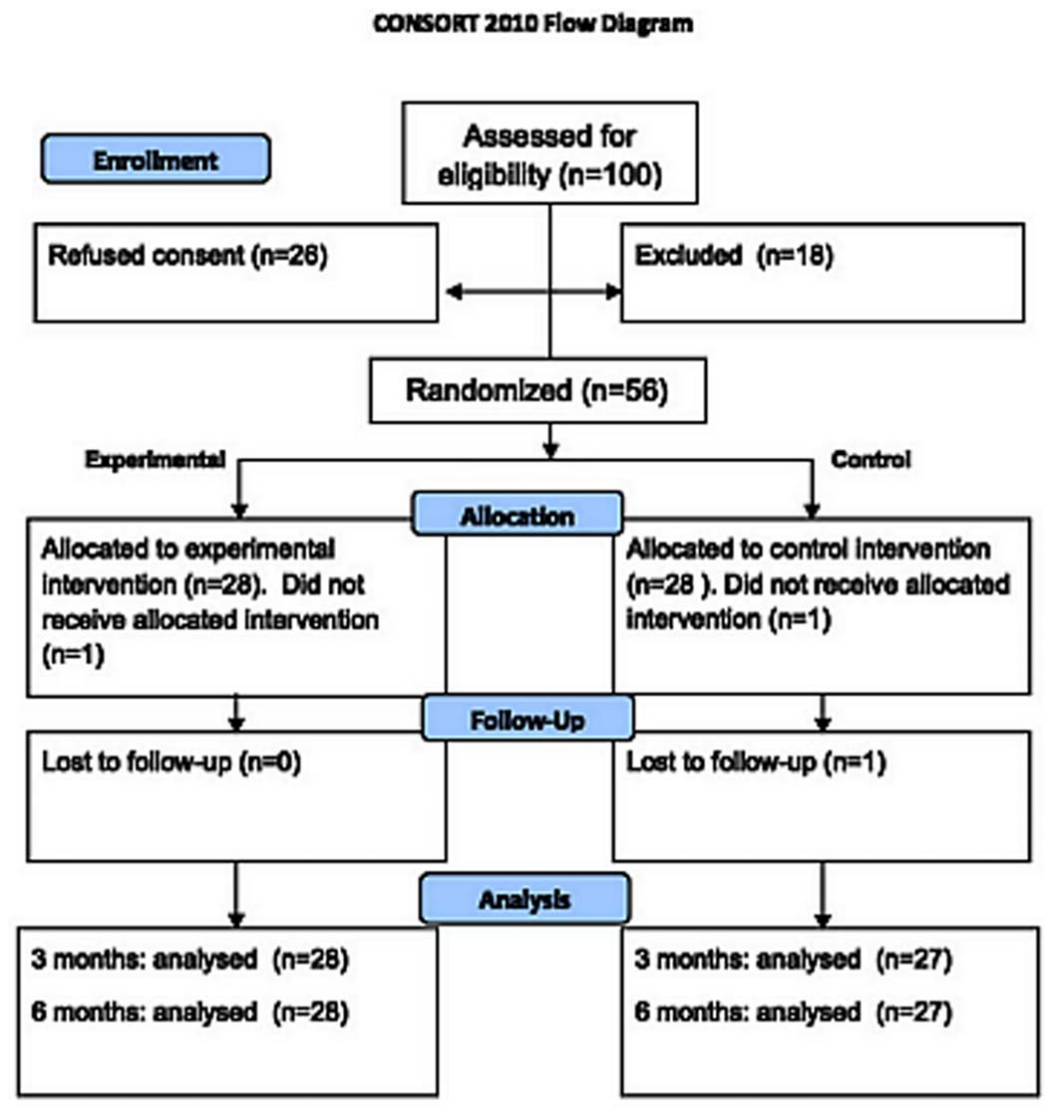
CONCERT flowchart.

100 families were eligible and 26 refused to participate in the trial (see Figure 1). 21 of these 26 children were boys and 5 were girls. The most common reasons for not wanting to participate were: not having time to participate in the groups; or not wanting the children to receive medication for their problems. 74 children were assessed, 18 children were excluded (17 boys and 1 girl). This left 56 children (39 boys, 17 girls) to be randomized in total. They were all of Danish ethnicity. The 18 children were excluded because of not fulfilling the diagnosis of ADHD, or had autism, psychosis, low IQ, or the child/parents not wanting to participate in the groups.

Table 1 shows the distribution of demographic data, DSM-IV diagnoses, and clinical variables in the two intervention groups. The two groups appear to be reasonably similar. Only 7.3% of the children were assessed with secure attachment patterns. The children and parents were included in one of four identical eight-week treatment programmes with 12–17 participants per programme. There were no systematic differences in results seen between these programmes. In the primary outcome ‘hyperactivity-impulsivity’ subindex the mean difference 3 and 6 months after start of treatment in the two intervention groups were 2.22 (95% CI: −4.34 to 8.98) and 1.84 (95% CI: −3.98 to 7.66) and insignificant (p = 0.40).

**Table 1:** Sociodemographic and clinical variables (N = 56).

	Experimental(N = 28)	Standard(N = 27)
**Sociodemographic:**		
Males No(%)	19 (67.8)	20 (74.1)
Age/year mean(SD)	10.6(1.29)	10.2(1.34)
**ADHD diagnoses:**		
ADHD-inattentive No(%)	10(35.7)	6(22.2)
ADHD-hyperactive/impulsive No(%)	0(0.0)	2(7.4)
ADHD-combined No(%)	16(57.1)	16(59.2)
ADHD NOS No(%)	2(7.1)	3(11.1)
**Other axis 1 disorders:**		
Oppositional defiant disorder No(%)	4(33.3)	4(40.0)
Anxiety disorder No(%)	4(33.3)	2(20.0)
Depressive disorder No(%)	1(8.3)	1(10.0)
*Tics and Obsessive Compulsive* *Disorder No(%)*	0(0.0)	1(10.0)
Enuresis No(%)	2(20.0)	2(20.0)
Stuttering	1(5.0)	0
**Attachment competences:**		
Secure No(%)	2(7.1)	2 (7.4)
Insecure/preoccupied No(%)	2(7.1)	1(3.7)
Insecure/dismissing No(%)	19(67.9)	20(74.1)
Disorganized/secure No(%)	0(0.0)	0(0.0)
Disorganized/insecure No(%)	5(17.9)	4(14.8)
**Intelligence quotient:**		
WISC verbal mean(SD)	93.9(15.7)	87.4(13.3)
WISC non-verbal mean(SD)	94.8(19.0)	88.9(10.5)
**ADHD problematic in the parents:**		
ASRS scores ≥4 (father) No(%)	6(28.6) (n = 21)	1(5.0)(n = 20)
ASRS score ≥4 (mother) No(%)	6(21.4)(n = 28)	6(24.0)(n = 25)

Two children were excluded a few days after the randomization, one of them because his mother did not want her child to receive central stimulating medication, and we were not allowed to obtain outcome assessment from this child. The other child and his parents did not want to participate in the treatment, but all his outcome assessments were obtained and this child is included in the analysis.

The outcome measure changed significantly over time for most outcome measures, but the time course did not differ significantly between the two intervention groups for any outcome measures (Tables 2 and 3). It appears from the three right hand columns in the Table 3, which show the p values of the main effect of the intervention and its interaction with t and with t^2^ that on no occasion did the time course of an outcome measure differ significantly between the two intervention groups. This was not altered if insignificant effects including the intervention indicator were removed from the model one at a time and the analysis each time repeated using the reduced model. An analysis not including the two protocols specified stratification variables (sex and co-morbidity) gave similar insignificant results.

**Table 2:** Mean, SD values, Mean differences and Confidence Intervals (C.I.) at baseline, 3 months, and 6 months by treatment group.

Outcome measure	Time/month	Experimental treatment			Standard treatment			Betweengroup	Confidence Interval
		N	Mean	SD	N	Mean	SD	Mean diff.	Difference – C.I.
**Executive functions**	0	26	12.00	4.49	27	12.48	4.53	−0.481	−2.969–2.006
	3	27	9.30	4.58	27	8.44	4.21	0.85	−1.551–3.254
	6	28	8.54	4.29	27	9.15	4.55	−0.612	−3.002–1.778
**Academic score**	0	24	25.71	14.54	26	25.31	11.86	0.401	−7.119–7.920
	3	24	20.13	15.15	26	17.88	10.11	2.240	−5.030–9.511
	6	26	21.04	11.98	27	21.52	12.56	−0480	−7.254–6.293
**Aggressiveness score**	0	27	17.59	18.03	27	27.85	24.25	−10.259	−21.928–1.410
	3	27	10.00	12.58	26	11.58	11.89	−1.577	−8.331–5.177
	6	28	10.50	12.41	27	12.78	12.25	−2.278	−8.949–4.394
**Emotional score**	0	27	20.37	15.11	27	17.89	15.25	2.481	−5.809–10.772
	3	27	17.26	11.25	26	13.04	12.31	4.221	−2.279–10.720
	6	28	16.79	12.09	27	14.44	12.51	2.341	−4.312–8.994
**Hyperactivity score**	0	27	20.70	11.38	27	24.70	14.05	−4.00	−10.982–2.982
	3	27	16.15	11.45	27	13.93	13.24	2.222	−4.538–8.982
	6	28	15.21	9.58	27	13.37	11.86	1.844	−3.977–7.664
**Peer r. score**	0	27	8.22	6.12	27	8.63	5.41	−0.407	−3.562–2.747
	3	27	5.44	5.00	26	4.81	4.48	0.637	−1.986–3.259
	6	28	4.86	4.58	27	5.37	5.51	−0.513	−3.247–2.221
**Social p. score**	0	27	10.33	6.34	27	11.52	11.52	−1.185	−4.842–2.471
	3	27	6.89	5.68	27	7.85	5.93	−0.963	−4.135–2.209
	6	28	8.57	6.00	27	9.56	6.76	−0.984	−4.437–2.469

**Table 3:** A mixed model analyses of the primary and the six secondary outcome measures (p-values).

Outcome measure (priority)	Sex	Co-morbidity	t	t^2^	Intervention-group (G)	G⋅t	G⋅t^2^
SQ (hyperactivity score)* ^)^ (primary)	0.0009	0.013	<0.0001	0.051	0.40	0.33	0.40
Academic score (secondary)	0.97	0.10	0.16	0.010	0.69	0.96	0.30
SQ (aggressiveness score) (secondary)	0.037	0.018	0.0013	0.003	0.50	0.79	0.58
SQ (emotional score)* ^)^ (secondary)	0.42	0.0051	0.043	0.83	0.14	0.94	0.62
SQ (peer score)* ^)^ (secondary)	0.31	0.074	<0.0001	0.056	0.55	0.39	0.76
SQ(social score)* ^)^ (secondary)	0.048	0.79	0.089	0.005	0.80	0.68	0.93
Executive score (secondary)	0.55	0.028	<0.0001	0.027	0.22	0.99	0.41

* )To fulfil the assumption of normally distributed values a square root transformation (SQ) was done prior to the mixed model analyses.

A mixed-model analysis of each outcome measure without the intervention indicator included in the model but with the latter augmented by CAI-binary, CAI-binary⋅t, and CAI-binary⋅t^^2^^ showed that on no occasion did the CAI-binary significantly influence the time course of an outcome measure. The same was found when the analysis was repeated but this time with all fixed effects involving the intervention indicator (see Table 3) retained in the model. Corresponding analyses of ASRS (father)-binary and of ASRS (mother)-binary gave similar insignificant results.

We did not observe any adverse event during or following the experimental intervention or the control intervention.

## Discussion

The neutral results in this SOSTRA trial, where the difference in mean in the primary outcome showed insignificant results (p = 0.40) (mean value in the 3 month outcome 2.22 (95% CI: −4.34 to 8.98) and mean value in the 6 month outcome 1.84 (95% CI: −3.98 to 7.66)), is in accordance with the findings in our Cochrane review as well as with the conclusions of the meta-analyses performed by Kavale et al. and Van der Oord et al. [19]; [20]; [23] but seems to differ from the results of de Boo and Prins and Majewicz-Hefley [21]; [22].

However, both of these latter meta-analyses had no evaluation of systematic errors (bias) in the trials included and the results are therefore questionable. We could not find support for our hypothesis that adding social skills training and parental training to the standard treatment would give a statistical significant difference on the children’s ADHD symptoms and social and emotional competences compared with the standard treatment alone.

One of the baseline findings in SOSTRA is especially interesting, as only 7.3% of the children had a secure attachment competence, as opposed to 61% in a normal population [45]. This has also been found in other studies [25]; [27]; [28] and supports the contention that there is an association between attachment problems and ADHD. Several studies show that insecure attachment competences in the small child is significantly associated with ADHD symptoms [25]–[27]. In the study by Clarke et al. they found that the nature of the attachment insecurity in the ADHD children were heightened emotional expressions; showing as strong out of control affects. The authors argue that treatment for ADHD must incorporate relationship-building components. Niederhofer and colleagues suggest that insecure attachment should be included in the list of problems associated with ADHD. Therefore, it may be speculated that these children need a form of treatment that focuses on their inability to form relationships and their social problems. There is a tendency towards more medication for children with ADHD and even if this treatment has a short-term effect, it is not addressed to alleviate social skills problems [10]. Social skills training are recommended as a part of a cognitive/behavioural treatment intervention regimen in both the European and the Nice Guidelines [17]; [18]. This recommendation must be discussed as there at the moment is no evidence for this treatment. However, absence of evidence is not evidence of absence of effect! There is a need for more research on this topic, and it seems like that there is necessary to develop another more profound type of social skills training. There is a need for another type of social skills training, which can help the children to deal with their attachment problems as well. This may mean a longer treatment programme, and a treatment that can change more profound aspects of the child’s personality. This treatment needs to focus on the cognitive aspect and also the affective.

In the SOSTRA trial we discovered a large effect over time for both the groups together, e.g., the children’s social problems scores, aggressiveness, and hyperactivity scores showed highly significant changes towards fewer symptoms (Table 3). We cannot state anything about the reason for this, apart from the fact that this development reflects the intervention effects of standard treatment as well as regressions towards the mean. Our SOSTRA trial has several limitations. Most important is the small number of participants. Based on our sample size calculation and our decision on a clinical relevance, we did not find any significant effects of social skills training. If more patients had been included we might have been able to discover smaller significant effects. We used a beta of 80%, which gives a 20% chance for a type 2 error. We cannot exclude that such an error may have occurred. Another limitation is the use of teacher-rated measurement scales. The teachers might not be able to track potential small changes in the children’s symptoms in classes with 25 other children. Furthermore, the therapists who were responsible for the children during the experimental intervention were also (but not on the same day) responsible for the parent group in the control arm. It is possible that these therapists have transferred elements from the experimental treatment to the parent group receiving the control intervention consisting of standard treatment. Finally, some of the children moved to another school during the trial, so different teachers completed the outcome forms, resulting in random errors.

The strength of the SOSTRA trial is that we published the design protocol before we embarked on the trial [24]. We performed our sample size calculation based on the primary outcome measure, conducted a computer generated randomization procedure, and conducted a proper allocation concealment to reduce selection bias. Finally, to strengthen reliability, we videotaped our manual based interventions. Furthermore, we conducted blind outcome assessments, data management, and intention-to-treat analyses, and reported on all outcomes as stipulated in our protocol. Hereby we tried to minimize bias [46]–[48]. We also included a parent group that was designed to support the children’s group, giving the parents information about the topic that their children were working with, and also assuring the parents that the children could manage their homework in social skills training. Another strength of the SOSTRA trial was the measurement of attachment styles in children with ADHD.

### Conclusions

In accordance with our Cochrane systematic review on social skills training for children with ADHD, we found no significant benefit or harm in any of the outcome measures of the SOSTRA trial. This suggests that on the basis of our sample size calculation and our consideration of a necessary relevant effect size, currently, there is no evidence to recommend or reject social-skills training with or without parental training for ADHD children. This result and the fact that 93% of the children who were assessed by the Child Attachment Interview at baseline had a insecure attachment disorder, leads us to believe that there may be a need for more profound, longer lasting types of interventions that might result in a change in children’s ADHD symptoms, to improve their social and relational competence, and thereby avoid serious further development of the disease.

## Supporting Information

Checklist S1 **CONSORT Checklist.**
(DOC)

Protocol S1 **Trial Protocol.**
(DOC)
