# Supplementary material for: Social-Skills and Parental Training plus Standard Treatment versus Standard Treatment for Children with ADHD – The Randomised SOSTRA Trial
Source: PLoS One. 2012 Jun 20;7(6):e37280. doi: 10.1371/journal.pone.0037280 (PMC3380035; doi:10.1371/journal.pone.0037280)
Supplement: Protocol S1 — Trial Protocol. (DOC) [file pone.0037280.s002.doc]

| 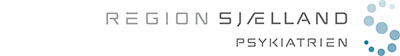  Social skills training and standard treatment versus standard treatment for children with ADHD - attention deficit hyperactivity disorder  *Attachment problems among children with ADHD*  SOSTRA-ADHD  Version 3.0  Version date June 22, 2009  Protocol reg. no. SOSTRA-BU/REGIONSJ.1  Clinical Trials.gov NCT00937469  Ethics Committee no. SJ-85  Data Protection Agency no. DO50892  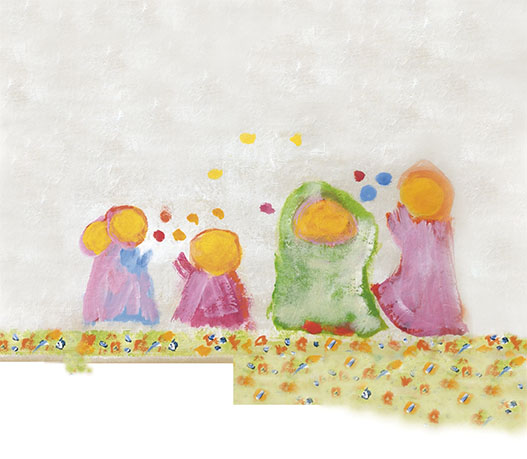 |
| --- |

# Table of contents

[Table of contents 2](#__RefHeading___Toc324542189)

[Abbreviation list 4](#__RefHeading___Toc324542190)

[Abstract 5](#__RefHeading___Toc324542191)

[Null hypotheses 6](#__RefHeading___Toc324542192)

[Organization and collaborators: 7](#__RefHeading___Toc324542193)

[Background 9](#__RefHeading___Toc324542194)

[Treatment of ADHD 10](#__RefHeading___Toc324542195)

[Pharmacological management of ADHD 10](#__RefHeading___Toc324542196)

[Psychosocial treatment forms 11](#__RefHeading___Toc324542197)

[Evidence from social skills training 11](#__RefHeading___Toc324542198)

[Parental work 11](#__RefHeading___Toc324542199)

[Design 12](#__RefHeading___Toc324542200)

[Ethics 12](#__RefHeading___Toc324542201)

[Participant information 12](#__RefHeading___Toc324542202)

[The conduct of the trial 12](#__RefHeading___Toc324542203)

[The purpose of the trial 12](#__RefHeading___Toc324542204)

[The design of the trial 12](#__RefHeading___Toc324542205)

[Randomization 13](#__RefHeading___Toc324542206)

[The intervention 13](#__RefHeading___Toc324542207)

[Experimental treatment 14](#__RefHeading___Toc324542208)

[Standard treatment 15](#__RefHeading___Toc324542209)

[Blinding 16](#__RefHeading___Toc324542210)

[Participants 16](#__RefHeading___Toc324542211)

[Inclusion criteria 16](#__RefHeading___Toc324542212)

[Exclusion criteria 16](#__RefHeading___Toc324542213)

[Inclusion period and participant selection 17](#__RefHeading___Toc324542214)

[Selection of patients 17](#__RefHeading___Toc324542215)

[The procedure of the assessment 17](#__RefHeading___Toc324542216)

[Procedure 18](#__RefHeading___Toc324542217)

[19](#__RefHeading___Toc324542218)

[Assessment instruments and effects measurements 19](#__RefHeading___Toc324542219)

[Experimental hypotheses 20](#__RefHeading___Toc324542220)

[Null hypotheses 20](#__RefHeading___Toc324542221)

[Outcome measurements 21](#__RefHeading___Toc324542222)

[Primary outcomes 21](#__RefHeading___Toc324542223)

[Secondary outcome 21](#__RefHeading___Toc324542224)

[1. Social Skills: measured by the Social Problems Scale (teacher rated) from Conners CBRS and by the Peer Relations Scale (teacher rated) subscore from Conners 3. 21](#__RefHeading___Toc324542225)

[Tertiary outcomes 21](#__RefHeading___Toc324542226)

[Compliance by therapists 21](#__RefHeading___Toc324542227)

[Medical protocol 22](#__RefHeading___Toc324542228)

[Statistical analysis plan 22](#__RefHeading___Toc324542229)

[Sample size 23](#__RefHeading___Toc324542230)

[Secondary outcomes 23](#__RefHeading___Toc324542231)

[Tertiary Outcomes 23](#__RefHeading___Toc324542232)

[Group comparison at entry 23](#__RefHeading___Toc324542233)

[Missing data 23](#__RefHeading___Toc324542234)

[Intention-to-treat principle and per-protocol analyzes 23](#__RefHeading___Toc324542235)

[Interim analysis and rules for changing end of trial 24](#__RefHeading___Toc324542236)

[Data storage 24](#__RefHeading___Toc324542237)

[Publications plan 24](#__RefHeading___Toc324542238)

[Funding 24](#__RefHeading___Toc324542239)

[Supplementary material 24](#__RefHeading___Toc324542240)

[References: 24](#__RefHeading___Toc324542241)

#

# Abbreviation list

**ADHD** = Attention Deficit Hyperactivity Disorder

**ASRS** = Adult ADHD Self-Report Scale

**CAI** = Children Attachment Interview

**DSM-IV** = Diagnostic and Statistical Manual of Mental Disorders

**ICD-10** = International Classification of Diseases

**IQ** = Intelligence Quotient

**K-SADS-PL** = Schedule for Affective Disorders and Schizophrenia for School-Age Children

Present and Lifetime version

**MTA** = Multimodal Treatment study

**SCQ** = Social Communication Questionnaire

**SDQ =** Strength and Difficulties Questionnaire

**SOSTRA** = Social Skills Training and Attachment

**TOVA** = Test of Variables of Attention

**WISC-III** = Wechsler Intelligence Scale for Children

**Conners CBRS** = Conners Comprehensive Behavior Rating Scales

**Conners 3** = Conners 3rd edition

# Abstract

Background:

Many children with ADHD, in addition to the main symptoms of hyperactivity, impulsivity, and a lack of ability to maintain attention, have difficulties with social interaction. This is a burden on the children’s relationships with parents, peers, and teachers and thereby influences their performance in school. Many children with ADHD find it very helpful to receive medical treatment, which has good effects on the children’s problems with hyperactivity, impulsivity, and inattention. However, social interaction problems are unlikely to be corrected by medication alone. Several studies have shown that social skills training can help children with ADHD in their social interactions. It will be helpful to further investigate treatment effects on the attachment styles of children with ADHD, as differences in attachment styles may influence the children’s relationships and social interaction problems.

The research intervention:

Social skills training consists of 8 weeks of group treatment with weekly sessions of one-and-a-half hours; training includes role playing, exercises, games, and homework, the latter of which includes the participants’ parents. At the same time, the parents participate in parental training groups that focus on supporting the children’s social training. The children’s and parents’ groups are led by two group therapists. The intervention is additional to the standard treatment, which all participants receive.

Control treatment:

The standard treatment consists of medical treatment, briefing, consulting, and supportive conversations with a focus on securing compliance with the treatments and on aiding children and their families with the difficulties resulting from the children’s illness. Furthermore, the parents participate in parents’ groups 3 times during the 8 weeks in which the experiment takes place. These groups last 2 hours and are managed by two nurses who work with the ADHD treatment group.

Method:

Children aged 8–12 years with diagnoses of ADHD are randomized either to standard treatment or to a combination of standard treatment, social skills training, and parental training. Sample size calculations show that 52 patients need to be included in the trial to show a 4-point difference in the primary outcome. Examinations of the children take place at baseline, after 3 months, and after 6 months.

Inclusion criteria:

The parents need to participate in parental groups at the Child Psychiatric Ambulant Unit in Holbæk. The patients (and their parents) need to understand and speak Danish to an extent where the examination and treatment can take place without an interpreter. The patients’ parents need to give written informed consent to participate in the trial. The children need to be 8–12 years old. Children need to have total IQ scores >80 on either the verbal or nonverbal WISC-III IQ indices. The children need to have a diagnosis of ADHD according to the criteria established by the DSM-IV.

Exclusion criteria:

Children with schizophrenia (paranoid, disorganized, catatonic, undifferentiated, residual, schizoaffective disorder, delusional disorder, brief psychotic disorder, shared psychotic disorder, or psychotic disorder not otherwise specified). Children with autism (autistic disorder, childhood disintegrative disorder, Asperger’s syndrome) or a score of 15 or less on either Social Communication Questionnaire (SCQ) scale. Violent and criminal youth. Patients who did not provide informed consent. Children who demonstrate resistance against participation in the trial. Children who have previously taken medication for ADHD.

Experimental hypotheses

1. There is a significant difference between the clinical effects of the combination of social skills training, parental training, and standard treatment versus standard treatment only at 3 and 6 months after the beginning of the treatment with respect to hyperactivity/impulsivity problems.

Measured by: “Hyperactivity/Impulsivity” subscore from Conners 3.

1. There is a significant difference between the clinical effects of the combination of social skills training, parental training, and standard treatment versus standard treatment only at 3 and 6 months after the beginning of the treatment with respect to the children’s social competences.

Measured by: “Social Problems” subscore from Conners CBRS and “Peer Relations” subscore from Conners 3.

1. There is a significant difference between the clinical effects of the combination of social skills training, parental training, and standard treatment versus standard treatment only at 3 and 6 months after the beginning of the treatment with respect to the children’s aggressive behavior and emotional distress.

Measured by: “Aggressive Behaviour” and “Emotional Distress” subscores from Conners CBRS.

1. There is a significant difference between the clinical effects of the combination of social skills training, parental training, and standard treatment versus standard treatment only at 3 and 6 months after the beginning of the treatment with respect to the children’s academic performance and executive functioning.

Measured by: “Academic Difficulties” subscore from Conners CBRS and “Executive Functioning” subscore from Conners 3.

1. There is a significant difference between the clinical effects of the combination of social skills training, parental training, and standard treatment versus standard treatment only at 3 and 6 months after the beginning of the treatment on the children’s social competences and symptoms of ADHD with respect to the different competences of attachment.

Measured by: Child Attachment Interview (CAI).

1. There is significant difference between the clinical effects of the combination of social skills training, parental training, and standard treatment versus standard treatment only at 3 and 6 months after the beginning of the treatment on the children’s social competences and symptoms of ADHD with respect to the parents’ level of ADHD symptoms.

Measured by: ASRS Symptom Checklist.

# Null hypotheses

- There is no significant difference between the clinical effects of the combination of social skills training, parental training, and standard treatment versus standard treatment only at 3 and 6 months after the beginning of the treatment.
- There is no difference in the effects of one or the other treatment in terms of the children’s different forms of attachment styles.
- There is no difference in the effects of one or the other treatment in terms of the children’s parents’ ADHD symptoms.

Primary outcomes

1. **ADHD symptoms: measured by the “Hyperactivity/Impulsivity Scale” (teacher rated) subscore from Conners 3.**

Secondary outcomes

1. **Social Skills**: measured by the “Social Problems Scale” (teacher rated) subscore from Conners CBRS and by the “Peer Relations Scale” (teacher rated) subscore from Conners 3.
2. **Aggressive behavior**: measured by the “Aggressive Behaviour Scale” (teacher rated) subscale from Conners CBRS.
3. **Emotional distress**: measured by the “Emotional Distress Scale” (teacher rated) subscale from Conners CBRS.
4. **Executive functions**: measured by the “Executive Functioning” (teacher rated) subscale from Conners 3.
5. **Academic performance**: measured by the “Academic Performance” (teacher rated) subscale from Conners CBRS.
6. **Overall psychopathology**: measured by the Conners CBRS Scale; T-score (teacher rated).
7. **Overall ADHD**: measured by the Conners 3 Scale; T-score (teacher rated).

Tertiary outcomes

1. **Social skills and symptoms of ADHD measured in relation to attachment style**: measured by CAI.
2. **Social skills and symptoms of ADHD measured in relation to parental ADHD symptoms**: measured by the ASRS Symptom Checklist.

Inclusion period

The study period for the trial will start in August 2009, and the last follow-up of the last participant will take place in November 2010.

# Organization and collaborators:

The project is a collaboration between Børne- og Ungdomspsykiatrisk Afdeling, Region Sjælland, Region Sjællands Forskningsenhed, and the Copenhagen Trial Unit.

Principal investigator: Psychologist Ole Jakob Storebø, who took the initiative for the project, is in charge of collecting research data during both examinations. Ole Jakob Storebø is registered as a PhD student at Sundhedsvidenskabeligt Fakultet, Københavns Universitet.

Parental groups and social skills training groups are managed by personnel employed at Børnepsykiatrisk Ambulatorium, Holbæk.

Main supervisor: Erik Simonsen, Associate professor. Københavns Universitet. Head of the Psychiatric Research Unit, Region Sjælland. Smedegade 10-16, 4000 Roskilde. E-mail: es@regionsjaelland.dk

Supervisors:

Jesper Pedersen, MD, PhD. Børnepsykiatrisk Ambulatorium Holbæk, Børne- og Ungdomspsykiatrisk Afdeling, Region Sjælland, Smedelundsgade 60, 4300 Holbæk. E-mail: jpee@regionsjaelland.dk

Per Hove Thomsen, professor, MD. Børne- og Ungdomspsykiatrisk Hospital, Århus Universitetshospital, Risskov, Harald Selmers vej 66, 8240 Risskov. E-mail: pht@buh.aaa.dk

Christian Gluud, MD. Leader of Copenhagen Trial Unit, Rigshospitalet**.** Blegdamsvej 9, 2100 København**.** E-mail: cgluud@ctu.rh.dk

# Background

A systematic review of a survey of the international prevalence of attention deficit hyperactivity disorder (ADHD) found that 5% of all children suffer from ADHD (Polanczyk & Rohde, 2007). The number of children who are referred to child psychiatric care with suspicion of ADHD is increasing internationally (BUP, 2008; Goldman et al., 1998; NICE, 2008; Swanson, 1995).

ADHD consists of 18 categories of symptoms in both diagnosis classification systems, the International Classification of Diseases (ICD-10) and the Diagnostic and Statistical Manual of Mental Disorders (DSM-IV) ([WHO, 1992](../WHO%201992); [APA, 1994](../APA%201994); [APA, 2000](../APA%202000)). However, there are different subdiagnoses of ADHD in DSM-IV, of which specific symptoms are identified. These subtypes are the “predominantly inattentive type” and the “predominantly hyperactive-impulsive type.” The most common subtype to be diagnosed among children and adults is combined type ADHD.

Many children with ADHD experience difficulties in social interaction, which is a burden in their relationships with parents, peers, and teachers. ADHD cannot be overcome using medication alone. Children with ADHD often have seriously disturbed relationships with peers, and they find it difficult to develop and maintain friendships (Whalen & Henker, 1985; Landau & Moore, 1991).

The major difficulties faced by children with ADHD symptoms in terms of social interaction with peers can be exacerbated by others’ negative reactions to their behavior. A vicious circle can therefore easily develop, and the difficulties can increase (Hoza et al., 2005). There seems to be a connection between lack of popularity during childhood and risk of involvement in criminality later in life (Roff et al., 1972). The learning difficulties of children with ADHD have serious effects on their performance in school; interventions that especially focus on improving school performance can be helpful in addressing this problem (Raggi & Chonis, 2006).

Social skills training is a method of intervention that can help children to improve their social competences. Social skills training aims to teach the child to regulate his/her behavior in relation to normal social rules. Learning takes place by means of role playing, exercises, games, and homework, which includes parents and possibly teachers. The training programs often include parental education and set the scene for the parents to actively train with the children in their own environment. A study by Grizenko et al. (2000) showed that the effects of social skills training were improved when training in competences related to social perspectives were included.

Attachment competences concern an infant’s relationship with his/her primary caretaker. The theory of attachment was devised by John Bowlby and Mary Ainsworth and was outlined in three articles by Bowlby (1958, 1959, 1960). The different styles of attachment that children can have are secure attachment, insecure dismissing, insecure preoccupied, and disorganized attachment. Bowlby’s theory of attachment has a biological focus, as he claimed that children look for attachment to their caretakers on the basis of their survival instinct. The child looks to the mother to provide safety when he/she feels fear or is hungry, etc. The theory also addresses aspects of development, because there are three types of motivation for learning and developing: attachment, exploration, and fear. The child’s experience with the caretaker causes the development of so-called “internal working models,” which reflect external experiences internally. These experiences could cause the development of a child with a secure attachment style in situations where the internal working models are primarily positive; if negative internal working models are predominant, these experiences could also cause the development of an insecure child (Bowlby, 1969).

A child with a secure attachment style can use his/her primary caretakers as a safe base from which he/she can go “out in the world” to take “discovery trips,” and later on, this child meets the outside world with positive expectations and trust. A child with an insecure attachment style has negative expectations of his/her surroundings, and his/her interactions with the environment may be marked by anxiety. It is important to stress that problems with attachment competences that a child can develop as a consequence of difficulties in his/her interactions with primary caretakers can also be caused by inborn traits of the child.

Fonagy (2006) claims that processes of attachment are most likely connected to the development of specific psychological functions or mechanisms that play essential roles in the organization of adequate behavior.

An ADHD diagnosis is not a sufficient basis to form a treatment plan for the specific child and his/her family. It is important to explore the severity and comorbidity of psychological, behavioral, and learning difficulties. The assessment of the child’s difficulties needs to be a part of a wider contextual evaluation of his/her family’s situation. Therefore, research is needed that focuses on changes in personality and on the child’s ability to relate to other people and other psychological functions. This is why it is relevant to examine the differences in treatment effects among ADHD children in relation to their attachment styles and competences.

Several studies have shown a connection between ADHD and disorganized attachment style. In a study by Punto et al. (2006) in which children were followed from the ages of 0–7 years, a clear connection was seen between disorganized attachment at an early age and the later development of ADHD symptoms. This finding leads to the conclusion that these children also need a form of treatment that focuses on their ability to form relationships and their social problems.

In clinical populations, the majority children diagnosed with ADHD are boys (Cantwell, 1996). In studies of the general population that included both genders, the difference in ADHD incidence rates between the sexes was considerably smaller, which indicates underdiagnosis of girls with ADHD (Brown et al., 1991; Carlson et al., 1997; Gaub & Carlson, 1997).

Disorders comorbid with ADHD often include behavioral disorders, depression, anxiety, tics, disturbances of motor skill development, learning difficulties, and verbal and cognitive difficulties (Jensen et al., 2001; Kadesjö et al., 2001).

## Treatment of ADHD

# Pharmacological management of ADHD

The effects of pharmacological treatment of children and adolescents with ADHD are well documented; medication is reported to have beneficial effects on major symptoms in the hyperactivity, impulsivity, and attention categories in about 80% of the patients treated ([Barkley, 1997](../Barkley%201997); [Gadow et al., 1990](../Gadow%201990); [Gillberg et al., 1997](../Gillberg%201997); [Gilmore & Milne, 2001](../Gilmore%202001); [Klassen et al., 1999](../Klassen%201999); [Malone et. al., 1993](../Malone%201993); [MTA, 1999](../MTA%201999a); [Spencer et al., 1996](../Spencer%201996)). The most common drugs used for the treatment of ADHD in children and adolescents are methylphenidate, atomoxetine, and dexamphetamine ([NICE, 2008](../NICE%202008)). The functional mechanisms of the medications are not clearly known, but it is presumed that their effects on ADHD symptoms are related to their stimulant effects on dopaminergic and noradrenergic neurotransmission in the central nervous system (CNS) (ADHD, [Socialstyrelsen Sverige, 2002](../Socialstyrelsen%20Sverige%202002)). Many children and adolescents with ADHD often have additional difficulties with language, learning, anxiety, and conflict-ridden interactions with parents/teachers, and there is little evidence that stimulant medications affect these outcomes ([Hinshaw, 1984a](../Hinshaw%201984a); [Hinshaw, 1984b](../Hinshaw%201984b); [Tannock, 2001](../Tannock%202001)). In addition to this, adverse reactions to these medications are a cause for concern, the most common being headaches, sleeping problems, tiredness, and problems with appetite, most of which stop when medication intake is discontinued. Serious adverse reactions affect 3–6% of children ([Pliszka, 1998](../Pliszka%201998); [Block, 1998](../Block%201998); [MTA, 1999a](../MTA%201999a); [Cherland, 1999](../Cherland%201999)). There is evidence that dexamphetamine affects children’s sleep and can cause dry mouth, thirst, decreased appetite, weight loss, and stomachaches and contributes to the risk of regressive, dependent behavior and psychosis ([NICE, 2008](../NICE%202008)). Atomoxetine is associated with pain, nausea, vomiting, decreased appetite, weight loss, dizziness, and slight increases in heart rate and blood pressure ([Wolraich, 2007](../Wolraich%202007)). There is also evidence that methylphenidate affects the children’s height and weight gain ([Schachar et al., 1997](../Schachar%201997); [Swanson et al., 2007](../Swanson%202007)). In addition, strong evidence exists for the connection between methylphenidate use and tics ([Palumbo et al., 2004](../Palumbo%202004)). Further, have there been reports of sudden death in children and adults treated with methylphenidate, but it is unclear whether these deaths are directly related to methylphenidate, and more research is being conducted on this topic ([US FDA, 2006](../US%20FDA%202006)).

# Psychosocial treatment forms

The best-documented nonmedical methods of treatment for ADHD are cognitive and behavioral. These methods consist of behavioral training, social skills training, cognitive training, and different forms of adjustment to the child’s difficulties with the environment at home and in school (Conners et al., 2001; MTA, 1999a). There is strong evidence for the effects of behavioral treatments in children with ADHD (Carr, 2000b). Cognitive treatments, on the other hand, have no clear evidence of effectiveness and cannot be recommended, as independent treatments or combined with medical treatments.

Several studies have provided clear results in this respect and have concluded that cognitive training added to standard medical treatment of ADHD has neither statistically nor clinically important effects on the main symptoms of ADHD or adaptation to home or school (Pelham et al., 1998).

# Evidence from social skills training

Several randomized, controlled studies have shown that social skills training helps children with ADHD. Social skills training teaches the child how to regulate his/her behavior vis-à-vis normal social rules. Learning in social skills training takes place by means of role-playing, exercises, games, and homework that involves parents and teachers. The programs include parental education and set the stage for parents to actively train with the child in his/her own environment. In Pfiffner and McBurnett’s randomized trial from 1997, in which social skills training and parental work (n = 27) or social skills training alone (n = 27) were compared to a control condition, a significant improvement in the children’s social skills was found in both treatment groups.

In a larger randomized trial (n = 100) by Tutty et al. (2003), a significant improvement in social skills was found after social skills training at home and in spare time, but not at school. In Tutty et al.(2003) a group of children with ADHD was offered 8 weeks of social skills training with parental groups plus medical treatment, whereas a control group received medical treatment only. Further, Antshel and Remer’s (2003) randomized trial showed an improvement in the children’s self-worth and ability for positive self-assertion. In that study, 120 children were randomly assigned to either social skills training plus medical treatment or medical treatment only.

Psychosocial forms of treatment, including social skills training, also have weaknesses, because they, like medical treatment, do not always seem to have long-term effects. Some children do not derive any benefits from social skills training; in some cases, this is because the parents are not involved in the treatment (ADHD, Socialstyrelsen, 2002). Some investigators have claimed that social skills training groups can have negative or reverse effects on children with behavioral problems, because many of the children’s aggressive and troubled behaviors can be problematic for learning social skills and can instead increase negative behavior (Mager et al., 2005). In a meta-analysis illustrating the effects of social skills training, it was found that social skills training offered alongside medical treatment had only 8% better effects on the ADHD children’s social problems than did medical treatment alone (Kvale et al., 1996). Nevertheless, larger beneficial effects have been described in studies in which social skills training is combined with parental training (Pfiffner & McBurnett, 1997; Frankel et al., 1997).

## Parental work

In the Børne- og Ungdomspsykiatrisk Selskabs reference program for assessment and treatment of children and youth with ADHD, reference is made to the fact that parental training can improve the parents’ caretaking praxis and to a certain extent ameliorate the child’s main ADHD symptoms (BUP, 2008).

Some studies in which parents filled out rating schedules have shown that parental training has limited effects when the mother herself has a high level of main symptoms of ADHD (Sonuga-Barke et al., 2001).

## Design

# Ethics

Participants will be informed of the trial in writing and by word of mouth, and written informed consent will be obtained from the participant’s guardian. The trial has obtained ethical approval and is registered with the Data Surveillance Agency and the clinicaltrials.gov registry.

This trial may confer an essential advantage to children with ADHD, because social skills training may have more significant effects on the social and emotional components of ADHD than medication can provide; furthermore, the connections between the different types of attachment competences and the effects of the social skills training can have beneficial influences on both the patients’ understanding and the treatment of the disease. There are no apparent ethical problems, because all of the participants are offered common medical treatment, and there are no known disadvantages of social skills training; however, any adverse events occurring during the intervention will be reported.

# Participant information

The participants in the trial were selected for recruitment because they were among the ADHD referrals received by the Børnepsykiatrisk Ambulatorium in Holbæk. The parents will first be informed about the project via a letter and a brochure, in which they will be offered the opportunity to participate in the trial. If they choose to participate, the investigators will meet with the child and the parents; at the meeting, the child will be assessed, and informed consent and participant information will be discussed. If the parents or the child does not want to participate in the study, the child will be placed on the normal waiting list for assessment. The parents will receive information about personal data protection, the purpose and method of the trial, possible disadvantages of participating, the goal of the research project (both for the individual child and for children affected by ADHD in general), the circumstances under which the entire trial can be interrupted, the nature of the standard treatment, where they can obtain further information about the trial, and a request to read the attached supplement, “The rights of participants in a biomedical research project.”

# The conduct of the trial

This trial will be conducted in accordance with this protocol, which has been approved by the Region Sjællands Ethical Committee the trial will employ good clinical practice and the best possible methods of avoiding accidents and systematic mistakes (bias). No changes will be made to the protocol without approval from the Region Sjællands Ethical Committee.

# The purpose of the trial

The primary purpose of the trial is to examine the effects of the combination of social skills training, parental training, and standard treatment versus standard treatment only in ADHD patients.

The secondary purpose of the trial is to examine how differences in the effects of treatment correlate with differences in attachment styles: secure attachment: insecure dismissing, insecure preoccupied, or disorganized attachment.

The tertiary purpose of the trial is to examine how differences in the effects of the treatment correlate with the degree of parents’ ADHD symptoms.

# The design of the trial

The trial is constructed as a two-armed, parallel-group, assessor-blinded trial. The participants are randomly assigned to conditions of either social skills training and parental training plus standard treatment or standard treatment only.

# Randomization

The randomization will be centralized and performed by computer software routines with hidden randomizing sequences; randomization will be performed by the Copenhagen Trial Unit (CTU), and only the data manager of CTU will have access to the coding. The primary investigator or the research secretary will call the CTU during normal business hours by dialing +45 35 45 71 71. After giving a personal identification code, patient number, stratification variable, sex, and describing the occurrence of comorbidity, the patient will be randomly assigned to either a combination of social skills training, parental training, and standard treatment or standard treatment only. Written informed consent will be obtained before randomization.

# The intervention

In total, 56 participants will be randomly assigned to the experimental intervention with standard treatment or standard treatment alone. The 56 participants will be divided into four identical treatment program sections. Below, the treatments in the two “arms” (conditions) are described; the start of medical treatment has been aligned vertically between the two groups.

**Arm 1:**

Social skills training =

Parental group =

Standard treatment parental group =

Standard treatment, e.g., medication =

**Arm 2:**

Standard treatment parental group =

Standard treatment e.g., medication =

**Arm 1:** Social skills training, parental group, and standard treatment for 8 weeks.

**Arm 2**: Standard treatment for 8 weeks.

Figure 1:

Arm 1: Experimental plus standard treatment for 8 weeks.

Arm 2: Standard treatment for 8 weeks.

### Experimental treatment

The group therapists participated in training courses before the start of the intervention to obtain adequate qualifications. The children are offered social skills training that will take place once a week for an hour and a half. During that time, the parents participate in parental groups. The intervention lasts a total of 8 weeks. There will be two therapists in each group. There will be four identical treatment programs, each lasting 8 weeks. There will be seven patients in each group and two groups in each treatment program. The treatment will be thoroughly described in a manual, and each session with the children will be recorded on video. The group sessions will be held on the same day of the week and at the same time for a firm structure, and the agenda will be the same every session. The manualized treatment program is organized on the basis of several randomized trials (Pfiffner & McBurnett, 1997; Antshel & Remers, 2003). Different methods of teaching the children social skills are used—methods that in several previous social skills programs have had good effects (Elliot & Gresham, 1993). Didactic instructions will be used, and tasks will include working with symbols (e.g., dolls), role-playing, different games, creative techniques, physical exercises, use of music, reading stories, playing games, and watching movies. Each session will have a theme. Examples of these are self-worth, nonverbal communication, feelings, management of aggression, and conflict resolution.

In the manual, the idea of “self/other perspective taking,” which easily lends itself to social skills training, is described. The aim is for the children to learn from other people’s opinions about the topic with which they work. However, it is important to accept and appreciate the children’s own opinions before they are challenged with offers of alternative ways to see things. It is necessary to work on creating a safe environment within the group, where the children feel safe enough to play and experiment. This way, the children can explore the understanding of everyone involved and of the different focus topics. The interventions need to be simple and clear.

The pedagogy in the group considers the special cognitive difficulties that the children have, so that the structure in each session is predictable, and the importance of the learning is emphasized. This is secured by regular items on the agenda that are written on the blackboard at each session:

- Opening round – what has happened since the last time?
- Revision from last time
- Homework from last time
- Presentation/education
- Participants working with topic
- Role playing/creative activities
- New homework
- Closing round

The therapists need to be clear, directive, and direct, but not confrontational or critical. Weight is attached to empathy, positive reinforcement, curiosity, and a “non-knowing,” mentalizing attitude. A relaxed atmosphere with room for humor is the aim.

In the parental groups, the themes from the children’s groups will be explained and discussed with the parents. Likewise, the children’s homework will be discussed with the parents.

### Standard treatment

Normal practice regarding ADHD patients on the Child Psychiatric Ambulant Unit in Holbæk:

The overall objective of the ADHD treatment is to secure “compliance,” which means that the team attaches importance to building an alliance with the family. The team creates safety, as the family needs sufficient counseling, psychoeducation, and support. The goal is for the entire family to become more confident and autonomous regarding the problems and challenges that an ADHD diagnosis presents to a child. After assessment and verification of ADHD diagnosis, the family is offered medical treatment for the child. The medical treatment always starts after a physical examination of the child. In the physical examination, the somatic condition of the child is examined, and an individually adapted neurological examination is performed. In some cases, “Movement ABC” (for children aged 4–8) and “Peeramid” (for children aged 9–14) are completed. The doctor orients the participants and their families about the advantages and disadvantages of medication. The family is asked to contact the Unit within 7 days of the first medication administration to discuss how the child is doing. This conversation serves to evaluate whether the dose needs to increase further. All children are examined again one month after the beginning of the treatment; positive effects and adverse effects are evaluated and pedagogical counseling is given. The somatic condition of the child is examined again; the extent of the adverse effects is evaluated; nutrition advice is given if the child’s weight has increased; and if there are problems regarding sleeping, the parents are offered a special duvet or medication that can rectify sleeping problems. The team is very attentive to side effects that medication could possibly rectify (for less complicated problems); when medication alone cannot address the side effects, the child is assessed further. The standard treatment involves a parent group in which up to 6 couples of parents meet 3 times during each of the 8 weeks of the experiment. In order to offer the parent group to all 12 couples of parents, the group is repeated 6 times during the 8 weeks of the study. This parent group ends at the same time as the experimental treatment groups. Besides general information about the disorder, the focus of the group is different ADHD-relevant topics, like the child’s relationships with siblings and peers. The group sometimes has an adult guest speaker with ADHD who tells the parents about life with an ADHD diagnosis and how it is to have children and perform everyday life functions with this disease. Furthermore, this group will serve as a network for the family, because the leaders of the group advocate mutual support among participants outside of the group sessions.

# Blinding

Participants, parents, treating doctors, and personnel in the Unit are not blinded to the randomized treatment assignments. However, the teacher who receives and fills out the schedules is blinded. To secure integrity, the primary investigator blinds the collected schedules, i.e., hides all data that could be used to identify the patient. All involved parties are instructed to not inform the children’s teachers of the interventions given. The blinded data will be transferred to the Copenhagen Trial Unit, which will manage data entry and statistical analysis.

# Participants

The Child Psychiatric Unit in Holbæk receives 300 children with suspected ADHD annually. The referrals come from general practice doctors, specialists, pedagogical and psychological advice centers in the municipality, psychologists in general practice, and others. The referrals are discussed during the visitation meetings of the department, after which the patients are put on a waiting list. Supplementary information is required, or the referral is rejected.

# Inclusion criteria

**1)** The parents need to take part in parental groups at Børnepsykiatrisk Ambulatorium in Holbæk.

**2)** The patients (and their parents) need to understand and speak the Danish language to an extent where a translator is not needed in order to be able to complete the assessment and treatment.

**3)** The patients’ parents need to give informed consent to participate in the trial.

**4)** The children need be 8–12 years old at the time of the start of the assessment.

**5)** Both boys and girls can participate.

**6)** Children need to have total IQ scores ≥80 on either the verbal or nonverbal WISC-III IQ indices.

**7)** The children must fulfill at least one of the following research criteria for the diagnosis of ADHD according to the DSM-IV (APA, 1994): 314.00, 314.01, 314.02, or 314.9.

**8**) The parents are required to understand the fact that their children need to receive medication for their ADHD.

# Exclusion criteria

Patients with the following diagnoses, according to DSM-IV:

**1)** Schizophrenia:

295.30 (Paranoid type)

295.10 (Disorganized type)

295.20 (Catatonic type)

295.90 (Undifferentiated type)

295.60 (Residual type)

295.70 (Schizoaffective Disorder)

297.1 (Delusional Disorder)

298.8 (Brief Psychotic Disorder)

297.3 (Shared Psychotic Disorder)

298.9 (Psychotic Disorder Not Otherwise Specified)

**2)** Children with autism, according to DSM-IV:

299.00 (Autistic Disorder)

299.10 (Childhood Disintegrative Disorder)

299.80 (Asperger’s Disorder)

or a cutoff score of >15 on one of the SCQ scales.

**3)** Violent and criminal youth.

**4**) Children with a total verbal and nonverbal IQ of <80 according to WISC-III.

**5)** Strong resistance from the child against participating in the trial.

**6)** Previously started medication for ADHD.

# Inclusion period and participant selection

The inclusion period for the trial is scheduled to start on August 1, 2009 and end on November 1, 2010.


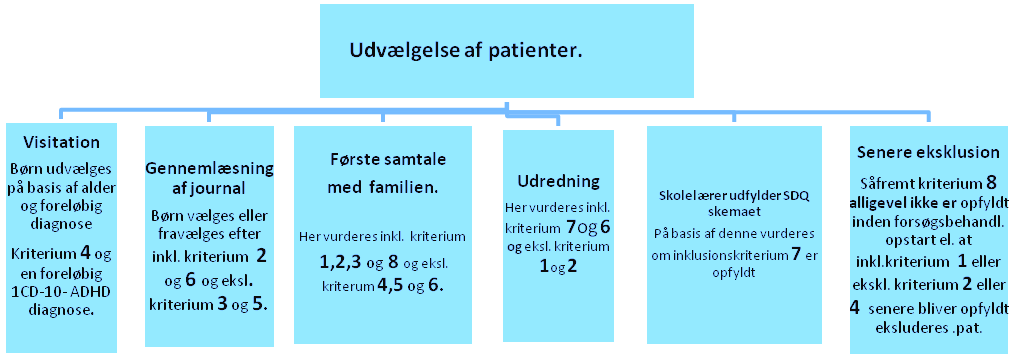


# Selection of patients

The charts of patients who have diagnoses of ADHD by ICD-10 resulting from the visitation meetings and who are 8–12 years old will be distributed to the principal investigator, who will read the papers in the charts. Here, inclusion criteria **2** and **6** and exclusion criteria **3** and **5** are evaluated. In cases in which all the inclusion criteria and none of the exclusion criteria are fulfilled, the child is included in the trial. If it is documented in the chart that some of the exclusion criteria are fulfilled, the child is excluded (for instance, if the child has been assessed before and has had an autism diagnosis). After this, the family will be contacted by mail, informed about the trial, and will receive a brochure describing the research project. Then, the families who choose to participate will be called in for assessment. They will receive a letter that gives concrete dates for single elements of the research assessment. If the family does not choose to participate, they will be called in for ordinary assessment later on.

# The procedure of the assessment

In the introductory conversation with the family, further information about the trial will be given, and the parents will be provided with information about the trial and a written informed consent form that needs to be filled out in order to participate in the trial. At this stage, inclusion criteria **1, 2,** and **3** and possibly exclusion criterion **4,** **5,** and **6** are fulfilled. Then, the primary investigator will complete diagnostic Schedule for Affective Disorders and Schizophrenia for School-Age Children Present and Lifetime version (K-SADS-PL) interviews with the parents and the child. These interviews will determine whether inclusion criterion **7** and exclusion criterion **1** are fulfilled. In this conversation, the child will also be screened for autism via two SCQ scales, which the parents need to complete. Here, exclusion criterion **2** will be evaluated, because the child will be excluded from the project if cutoff values of over 15 on one of these schedules are met. The parents also need to fill out the ASRS schedule, but the child will be included regardless of the results of this schedule.

After this, the primary investigator will have one of the child’s teachers fill out the Strength and Difficulties Questionnaire (SDQ). Here, inclusion criterion **7** will be evaluated. For inclusion in the study, a cutoff score of 6 on this scale needs to be met (Fletcher et al., 2001).

Children who have not been evaluated with the WISC-III test and children for whom the existing WISC-III test is older than 2 years will be tested with the WISC-III test by psychologists from the Unit. This test is used to evaluate inclusion criterion **6**.

The parents are informed in advance that if their child does not fulfill all inclusion criteria and no exclusion criteria, and cannot take part in the trial, assessment will still be provided by clinicians in the Unit. About 15–30 days before the beginning of the intervention treatment, the children with be tested using the CAI by the principal investigator. The child can be included in the trial regardless of the outcome of this test, because all attachment styles are acceptable for the study. After this, the child will be assessed with TOVA. The child will be observed in school by a consultant employed in the Unit. The TOVA test and the observations are used for clinical reasons; data from these were not included in the research project.

We count upon having 20 children who can be assessed when the inclusion period is scheduled to start. This number of participants is needed in order to end the process with approximately 14 participants who can be randomized. New programs with 20 new children will also be conducted in late autumn 2009, spring 2010, and autumn 2010 for a total of 4 identical treatment programs. All patients who are referred to the Child Psychiatric Unit in Holbæk with an ADHD diagnosis and who meet the age requirements will go through the above-mentioned evaluation process for possible participation in the trial.

# Procedure

The program itself is divided into several steps, each consisting of 5 boxes; 20 children with ADHD are assessed each time the program is run. In the following month, the children will be evaluated by a doctor and prescribed medication. The month after that, the medication will be adjusted and regulated so that all children have been put onto medication within the same month. In this last month before the experimental treatment begins, the children will be randomly assigned to either the experimental or the control treatment, and 14 days before the treatment begins, parents and teachers fill out the Conners CBRS (teacher rated) and Conners 3 (teacher rated) scales that form the baseline of the project. These schedules will also be filled out as the outcome measurements 3 and 6 months after the beginning of the treatment.

Assessment

of about 20 ADHD patients

Medical treatment.

Adjusting medicine. Baseline measurements plus CAI. Randomization of 12 patients.

Therapy versus standard treatment.

Therapy versus standard treatment.

**Outcome**

3 months

**Outcome**

6 months

1 2 3 4 5

The steps sketched above are repeated 4 times staggered at 2–3-month intervals. This means that the last outcome measurements will be made approximately at the beginning of 2011.

**2009 2010 2011**

**Sept Oct Nov Dec Jan Feb March April May June July Aug Sept Oct Nov Dec Jan Feb March Apr May June_July__**

##

## Assessment instruments and effects measurements

- **CAI** (Target et al., 2003; Shmueli-Goetz et al., 2008): The CAI focuses on the child’s experiences of his/her own present relevant relationships and measures the child’s view of the accessibility and sensitivity of his/her attachment figures through exploration of inner object representations. The test consists of 19 questions in which the child is asked to recall experiences with his/her important attachment figures, especially at times when the child has been sad, anxious, or sick. The interviews will be recorded on video, and both verbal and nonverbal statements will be scored. As a result of this test, the children are categorized as having one of the following attachment styles: secure attachment, insecure dismissing, insecure preoccupied, or disorganized attachment. The CAI can be used for children aged 7–13 years (Danish version).
- **K-SADS-PL.** This scale was translated into Danish by Dorthe Janne Petersen and Niels Bilenberg. This internationally known diagnostic interview system, the Schedule for Affective Disorders and Schizophrenia for School-aged Children, Present and Lifetime Version (K-SADS-PL), is an interview used to diagnose children and youths aged 6–18 years. Using an interview makes it possible to classify child and youth psychiatric diagnoses according to the DSM-III-R and DSM-IV systems.
- **Conners CBRS Teacher.** This schedule was translated into Danish by Ole Jakob Storebø, Kirsten Bach, Dorte Damm, Per Hove Thomsen, and Dansk Psykologisk Forlag. It is an internationally approved instrument that is used on children aged 6–18 years. The instrument has strong psychometric properties and measures behavior, school performance, and emotional and social competences.
- **Conners 3 Teacher.** This schedule was translated into Danish by Ole Jakob Storebø, Kirsten Bach, Dorte Damm, Per Hove Thomsen, and Dansk Psykologisk Forlag. It is an internationally approved instrument that is used on children aged 6–18 years. The instrument has strong psychometric properties and measures ADHD core symptoms, behavior, and emotional and social competences.
- **ASRS Symptom Checklist.** This is an instrument that assesses the 18 DSM-IV-TR criteria for ADHD. It is used on adults. From the American version of the Checklist, 6 of the 18 questions have been identified as the most predicative of ADHD. These six questions form the basis of the ASRS version 1.1 screening instrument and comprise part A in the symptom checklist. Afsnit B of the symptom checklist contains the remaining 12 questions (Danish version).
- **SCQ** (parent rated) is a screening instrument for evaluation of autism and autism spectrum disorders among children 4 years of age and older.
- **SDQ** (teacher rated) is a brief behavioral questionnaire for children ranging 3–16 years of age.
- **WISC-III** is a test that is used to evaluate intelligence and cognitive functioning among children aged 6–16 years.

# Experimental hypotheses

1. There is a significant difference between the clinical effects of the combination of social skills training, parental training, and standard treatment versus standard treatment only at 3 and 6 months after the beginning of the treatment with respect to hyperactivity/impulsivity problems.

Measured by: Hyperactivity/Impulsivity subscore from Conners 3.

1. There is a significant difference between the clinical effects of the combination of social skills training, parental training, and standard treatment versus standard treatment only at 3 and 6 months after the beginning of the treatment with respect to the children’s social competences.

Measured by: Social Problems subscore from Conners CBRS and Peer Relations subscore from Conners 3.

1. There is a significant difference between the clinical effects of the combination of social skills training, parental training, and standard treatment versus standard treatment only at 3 and 6 months after the beginning of the treatment with respect to the amount of children’s aggressive behavior and the children’s emotional distress.

Measured by: Aggressive Behaviour and Emotional Distress subscores from Conners CBRS.

1. There is a significant difference between the clinical effects of the combination of social skills training, parental training, and standard treatment versus standard treatment only at 3 and 6 months after the beginning of the treatment with respect to the children’s academic performance and executive functioning.

Measures: Academic Difficulties subscore from Conners CBRS and “Executive Functioning” subscore from Conners 3.

1. There is a significant difference between the clinical effects of the combination of social skills training, parental training, and standard treatment versus standard treatment only at 3 and 6 months after the beginning of the treatment with respect to the children’s social competences and symptoms of ADHD in relation to the different attachment styles

Measured by: CAI.

1. There is a significant difference between the clinical effects of the combination of social skills training, parental training, and standard treatment versus standard treatment only at 3 and 6 months after the beginning of the treatment with respect to the children’s social competences and symptoms of ADHD in relation to the parent’s amount of ADHD symptoms.

Measured by: ASRS Symptom Checklist.

# Null hypotheses

- There is no significant difference between the clinical effects of the combination of social skills training, parental training, and standard treatment versus standard treatment only at 3 and 6 months after the beginning of the treatment.
- There is no significant difference between the effects of one or the other treatment in terms of the children’s different attachment styles.
- There is no significant difference between the effects of one or the other treatment in terms of the children’s parents’ ADHD symptoms.

# Outcome measurements

All outcomes will be assessed before the intervention starts, after 3 months, and after 6 months.

# Primary outcomes

**1. ADHD symptoms: measured by the Hyperactivity/Impulsivity Scale (teacher rated) subscore from Conners 3.**

# Secondary outcome

# 1. Social Skills: measured by the Social Problems Scale (teacher rated) from Conners CBRS and by the Peer Relations Scale (teacher rated) subscore from Conners 3.

**2 Aggressive behavior**: measured by the Aggressive Behaviour Scale (teacher rated) subscore from Conners CBRS.

**3 Emotional distress**: measured by the Emotional Distress Scale (teacher rated) subscore from Conners CBRS.

**4 Executive functions**: measured by the Executive Functioning (teacher rated) subscore from Conners 3.

**5 Academic performance**: measured by the Academic Performance (teacher rated) subscore from Conners CBRS.

**6 Overall psychopathology**: measured by the Conners CBRS scale; T-score (teacher rated).

**7 Overall ADHD**: measured by the Conners 3 scale; T-score (teacher rated).

# Tertiary outcomes

1. **Social skills and symptoms of ADHD measured in relation to attachment style**: measured by CAI.
2. **Social skills and symptoms of ADHD measured in relation parental ADHD symptoms**: measured by the ASRS Symptom Checklist.

# Compliance by therapists

To secure compliance to the trial registration protocol, schedules for compliance with the interventions are implemented. Registration schedules are used to assure that planned material from the intervention is being sufficiently implemented. To secure compliance, the group therapists are monitored by an external supervisor with expertise in ADHD and social skills training.

# Medical protocol

Medical treatment for children with ADHD in the Unit is built upon official, evidence-based guidelines for such treatment. The medication that all children regularly receive needs to be recorded in the patients’ charts. All children in the trial need to be stabilized in terms of medication treatment before the treatment part of the experiment begins, and they need to take the medication for the entire trial period, i.e., 6 months after baseline measurement. The children will stay in the trial in spite of discontinuation of medication if the medication has been taken for a minimum of 2 months.

The family is asked to contact the Unit within 7 days of the first medical treatment to discuss how the child is doing. This conversation serves to evaluate whether the dose needs to increase further.

## Statistical analysis plan

Statistical analysis will be conducted by the Copenhagen Trial Unit (CTU) after the development of the trial database by the primary investigator in collaboration with the CTU’s data manager.

Before data from the trial are analyzed, an explicit statistical analysis plan will be developed by the trial statistician and the primary investigator. To ensure that the distributional prerequisites of the planned analyses of the primary outcomes are fulfilled, we could institute a “blind review” of the distribution of the primary outcomes before the statistical analysis plan is finished.

# Sample size

The sample size is calculated on the basis of type I error of 5% (α), type II error of 20% (β) (a power of 80%), and an allocation ratio of 1:1. In order to establish a clinically relevant difference between the intervention group and the control group with an effect size difference of 4 on the Conners rating scale’s Hyperactivity subscore (CBRS; this is one of the primary outcomes) and an assumed standard deviation of 5 on the same scale (Horns et al., 1991), a sample size of 26 participants in each group is necessary. This corresponds to a total of 56 participants to be randomized, under the assumption that 4 (8%) of the participants will not complete the trial (Antshel & Remer, 2003).

# Secondary outcomes

The primary outcomes (the differences in the Social Problems subscore from Conners CBRS and the Peer Relations subscore from Conners 3) will be analyzed via parametric testing if the data are normally distributed. The results will be presented with 95% confidence intervals and *P*-values assessed at significance levels of 5% with correction for multiple comparisons according to Holm-Bonferroni (Holm, 1979).

The secondary outcomes (the differences in the Aggressive Behaviour, Emotional Distress, and Academic Difficulties subscores from the Conners CBRS, the Executive Functioning (teacher rated) subscore of the Conners 3, and the total Conners CBRS and Conners 3 T-scores) will be analyzed via parametric testing if the data are normally distributed. The results will be presented with 95% confidence intervals and *P*-values assessed at significance levels of 5% with correction for multiple comparisons according to Holm-Bonferroni (Holm, 1979).

# Tertiary Outcomes

The tertiary outcomes (the differences in the primary outcomes categorized according to the four different attachment styles) will be analyzed in a linear fixed-effects model with the effect of attachment as a fixed factor and the five primary outcomes as dependent factors. The results will be presented with 95% confidence intervals and *P*-values assessed at significance levels of 5%.

# Group comparison at entry

To establish whether participants’ characteristics at entry to the trial are relatively similar between the 2 intervention groups (thereby conferring a low risk of confounding and selection bias), demographic data (sex and age) and other factors that can be expected to have an impact on the primary outcomes will be presented in a table of the participants’ entry characteristics.

# Missing data

Missing data will be handled in the analyses with imputation. If outcomes resulting in missing data (missing follow-up, retraction of informed consent, drop-out) are present, then the pattern of the missing data and the assumption of randomness (MAR = missing at random) in the missing data are investigated. If MAR is a plausible interpretation, a multiple imputation is performed with a multiple imputation analysis (MIA) computer software program. If MAR is not the plausible interpretation, the need for MNAR (missing not at random) will be analyzed in sensitivity analyses.

# Intention-to-treat principle and per-protocol analyzes

The statistical analysis of the outcomes will proceed according to the “intention-to-treat” principle, i.e., all participants randomly assigned to treatment groups will be included in the analysis in their randomly assigned intervention groups, irrespective of how much of the intervention they receive.

Per-protocol analyses will also be conducted for participants who comply with 50% or more of their assigned interventions.

With regard to the ICH Harmonized Tripartite Guideline, Statistical Principles for Clinical Trials E9 Analysis of Drug Trials, the analyses will first be conducted without adjustment for stratification variables, and then they will be repeated with adjustment for stratification variables.

# Interim analysis and rules for changing end of trial

There is no planned interim analysis.

All adverse events will be recorded with their frequency and cause. In case of an unacceptable high level of adverse events that are related to the intervention, the trial will be stopped.

# Data storage

The research data will be stored in locked cabinets in a locked office and handled according to Danish law on personal data. The data will be stored until October 31, 2011, and after that, it will be anonymized or destroyed.

# Publications plan

The following four articles are planned:

1) A Cochrane Review on social skills training for children with ADHD will be sent for admission to *Cochrane Library.* Title: **Social skills training for children with attention deficit hyperactivity disorder (ADHD).** (Protocol submitted).

2) An article about psychosocial forms of treatment for children with ADHD will be sent to *Nordic Psychiatry*. Title: **Psychosocial treatment for children with attention deficit hyperactivity disorder (ADHD).**

3) The results from the randomized examination will be sent to *Pediatrics* or *The Lancet.* Title: **Social skills training for children with attention deficit hyperactivity disorder (ADHD)**: **A randomized controlled clinical trial.**

4) An article to *Journal of Attachment*. Title: **Attachment Disorders in children with attention deficit hyperactivity disorder (ADHD).**

# Funding

The project received financial backing from Region Sjællands Universitets Sygehus (RESUS) in June 2008.

Total funding has been 333000 for 3 years.

Region Sjællands Forskningsfond granted 60000 to a Ph.D. stipend for the first year, 64000 to payment of practitioners, and 58000 to courses for the practitioners of the project. Fru C. Hermansen’s bursary granted 20000 to the project’s new training courses. Supervision, randomization, statistical analyses, and purchase of test materials were funded by Regions Sjællands Forskningsfond.

# Supplementary material

# References:

ADHD, Socialstyrelsen, Sverige-2002

Antshel KM. & Remer R. (2003) Social skills training in children with attention deficit hyperactivity disorder: A randomized-controlled clinical trial. *Journal of Clinical Child and Adolescent Psychology.* 32(1):153-165.

APA (1994) Diagnostic and Statistical Manual of Mental Disorders (DSM-IV). Fourth edition. Vol. IV. Washington DC: American Psychiatric Association

APA (2000) Diagnostic and Statistical Manual of Mental Disorders. Fourth Edition - Text revision edition. Vol. DSM-IV-TR. Washington DC: American Psychiatric Association.

Barkely RA. (1997) A review of stimulant drug research with hyperactive children. *Journal of Child Psychology and Psychiatry.* 18(2): 137-65.

Block S. (1998) Attention deficit disorder. In: *Child and Adolescent Psychopharmacology. The paediatric clinics of North America,* Editors: Findling R., Blumer J., London: W.B. Saunders Company. 1998.

Bowlby J. (1958) The nature of the child’s tie to his mother*. International Journal of Psycho-Analysis*. 39:1-23.

Bowlby J. (1959) Separation anxiety. *International Journal of Psycho-Analysis*. 41:1-25.

Bowlby J. (1960) Grief and mourning in infancy and early childhood. *The Psychoanalytic Study of the Child.* 15:3-39.

Bowlby J. (1969) *Attachment and Loss,* Vol. Attachment. London: Hogarth Press and the Institute of Psycho-Analysis.

Brown RT., Madanswain A., Baldwin K. (1991) Gender differences in a clinic-referred sample of attention-deficit-disordered children. *Child Psychiatry & Human Development*. 22(2):111-28.

BUP (2008)Børne- og Ungdomspsykiatrisk Selskabs reference program for ADHD.

Carlson CL., Tamm L., Gaub M. (1997) Gender differences in children with ADHD, ODD, and co-occurring ADHD/ODD identified in a school population. *Journal of the American Academy of Child and Adolescent Psychiatry*. 36(12):1706-14.

Carr A. (2000b) What works with children and adolescents? A critical review of psychological interventions with children, adolescents, and their families. London: Routledge.

Cantwell DP. (1996) Attention deficit disorder: a review of the past 10 years. *Journal of the American Academy of Child and Adolescent Psychiatry*. 35(8).

Cherland E. & Fritzpark R. (1999) Psychotic side effects of psychostimulants: A 5-year review. Canadian Journal of Psychiatry, 44:811-3.

Conners CK., Epstein JN., March JS. (2001) Multimodal treatment of ADHD in the MTA: An alternative outcome analysis. *Journal of American Academy of Child and Adolescent Psychiatry*. 40(2):159-67.

Elliot SN. & Gresham FM. (1993) Social skills interventions for children. *Behaviour Modification*. 17:287-313.

Fonagy P. (2006) *Tilknytningsteori og psykoanalyze.* oversat af Bjørn Nake fra engelsk efter attachment theory and psychoanalysis. København: Akademisk Forlag.

Fletcher J., Tannock R., Bishop DVM. (2001) Utility of brief ratings scales to identify children with educational problems. *Australian Journal of Psychology*. 53(2):63-71.

Frankel F., Myatt R., Cantwell DP., Feinberg DT. (1997) Parent-assisted transfer of children´s social skills training: Effects on children with and without attention-deficit hyperactivity disorder. *Journal of the American Academy of Child and Adolescent Psychiatry*. 36:1056-64.

Gadow KD., Nolan EE., Sverd J., Sprafkin J., Paolicelli L. (1990) Methylphenidate in aggressive-hyperactive boys: Effects on peer aggression in public school settings. *Journal of the American Academy of Child and Adolescent Psychiatry.* 29(5):710-8.

Gaub M., Carlson CL. (1997) Gender differences in ADHD: A meta-analysis and critical review [published erratum appears in *Journal of the American Academy of Child and Adolescent Psychiatry*. 36(12):1783]. *J Am Acad Child Adolesc Psychiatry*. 36(8):1036-45.

Gilberg C., Melander H., von Knorring A., Janols L., Thernlund G., Hagglof B., Eidevall-Walin L., Gustafsson P., Kopp S. (1997) Long-term stimulant treatment of children with attention-deficit hyperactivity disorder symptoms. A randomized, double blind, placebo-controlled trial. *Archives of General Psychiatry.* 54:857-64.

Gilmore A. & Milne R. (2001) Methylphenidate in children with hyperactivity: Review and cost-utility analysis. *Pharmacoepidemology and Drug Safety*. 10(2):85-94.

Goldman LS., Genel M., Bezman RJ., Slanetz PJ. (1998) Diagnosis and treatment of attention-deficit disorder in children and adolescents. *Journal of the American Medical Association*. 279:1100-7.

Grizenko N., Zappitelli M., Langevin JP., Hrychko S., El-Messidi A., Kaminester D., Pawliuk N., Stepanian MT. (2000) Effectiveness of a social skills training program using self/other perspective-taking: A nine. month follow-up. *American Journal of Orthopsychiatry*. 70(4)501-509

Hinshaw S., Henker B., Whalen C. (1984a) Cognitive-behavioral interventions for hyperactive boys; Comparative and combined effects. *Journal of Clinical Psychology*. 52:739-49.

Hinshaw S., Henker B., Whalen C. (1984b) Self control in hyperactive boys in anger inducing situations: effects on cognitive behavioural training and of methylphenidate. *Journal of Abnormal Child Psychology.* 12:55-77.

Holm S. (1979) A simple sequentially rejective multiple test procedure. Scandinavian Journal of Statistics, theory and applications. (6) 5-70.

Horns WF., Ialongo NS., Pascoe JM., Greenberg G., Packard T., Lopez M., Wagner A., Puttler L. (1991) Additive effects of psychostimulants, parent training, and self-control therapy with ADHD children. *Journal of the American Academy of Child and Adolescent Psychiatry*. 30(2):233-40.

Hoza B., Gerdes AC., Mrug S., et al. (2005) Peer assessed outcomes in the multimodal treatment of children with attention deficit hyperactivity disorder. *Journal of Clinical Child Adolescence Psychology*. 34(1):74-86.

Jensen PS., Hinshaw SP., Kraemer HC., et al. (2001) ADHD comorbidity findings from the MTA study: Comparing comorbid subgroups. *Journal of the American Academy of Child and Adolescent Psychiatry*. 40(2):147-58.

Kadesjö B., Gillberg C. (2001) The comorbidity of ADHD in the general population of Swedish school-age children. *Journal of Child Psychology and Psychiatry*. 42(4):487-92.

Kavale KA, Mathur SR, Forness SR, Rutherford RB, Quinn MM. Effectiveness of social skills training for students with behavior disorders: A meta-analysis. Advances in learning and behavioral disabilities 1997;11:1-26.

Klassen A., Miller A., Raina P., Lee SK., Olsen L. (1999) Attention-deficit hyperactivity disorder in children and youth: a quantitative systematic review of the efficacy of different management strategies. *Canadian Journal of Psychiatry*. 44(10):1007-16.

Jensen PS., Hinshaw SP., Kraemer HC. et al. (2001) ADHD comorbidity findings from the MTA study: Comparing comorbid subgroups. *Journal of the American Academy of Child and Adolescent Psychiatry.* 40(2):147-58.

Mager W., Milich R., Harris MJ. et al. (2005) Interventions group for adolescents with conduct problems: is aggression harmful or helpful? *Journal of Abnormal Child Psychology.* 33(3):349-62.

Malone MA., Swanson JM. (1993) Effects of methylphenidate on impulsive responding in children with attention deficit hyperactivity disorder. *Journal of Child Neurology*. 8(2):157-63.

Landau S & Moore LA (1991) Social skill deficit in children with attention-deficit hyperactivity disorder. *School Psychology Review*, 20:235-51

MTA (1999) MTA Cooperation Group. A 14 month randomized clinical trial of treatment strategies for attention deficit hyperactivity disorder. *Archives of General Psychiatry*. 56:1073-86.

NICE (2008) National Collaborating Centre for Mental Health commissioned by the National Institute for Health and Clinical Excellence. Attention deficit hyperactivity disorder. Diagnosis and management of ADHD in children, young people and adults. National Clinical Practice Guideline Number 72.

Palumbo D., Spencer T., Lynch J., Co-Chien H., Faraone SV (1994) Emergence of tics in children with ADHD: Impact of once-daily OROS® methylphenidate therapy. *Journal of Child and Adolescent Psychopharmacology.* 14(2):185.94.

Pelham WE. Jr., Wheeler, T., Chronis, A. (1998) Empirically supported psychosocial treatments for attention deficit hyperactivity disorder. *Journal of Clinical Child Psychology*. 27(2):190-205.

Pfiffner LJ. & McBurnet K. (1997) Social skills training with parent generalization: Treatment effects for children with attention deficit disorder. *Journal of Consulting and Clinical Psychology.* 65(5)749-57.

Polanczyk G., Rohde LA. (2007) Epidemiology of attention-deficit/hyperactivity disorder across lifespan. *Current Opinion in Psychiatry*. 20(4):386-92.

Pliszca (1998) The use of psychostimulants in the paediatric patient. In: *Child and Adolescent Psychopharmacology. Vol 45 (The Pediatric Clinics of North America)*, Editors: Findling R., Blumer J. 1998. London: W.B. Saunders Company.

Punto C., Turton P., Hughes P., White S., Gillberg C. (2006) ADHD and infant disorganized attachment-A prospective study of children next-born after stillbirth. *Journal of Attention Disorder.* 10(1)83-91.

Raggi VL. & Chonis AM. (2006) Interventions to address the academic impairment of children and adolescent with ADHD. *Clinical Child and Family Psychological Review*. 9(2): 85-111.

Roff M., Sells SS., Golden, MM. (1972) Social adjustment and personality development in children. Minneapolis: University of Minnesota.

Schachar, RJ., Tannock R., Cunningham C., et al. (1997) Behavioral, situational, and temporal effects of treatment of ADHD with methylphenidate. *Journal of the American Academy of Child and Adolescent Psychiatry*. 36, 754-763

Shmueli-Goetz Y., Target M., Fonagy P., Datta A. (2008) The child attachment interview: A psychometric study of reliability and discriminant. *Developmental Psychology.* 44(4)939-956.

Sonuga-Barke EJ., Daley D., Thompson M., Laver-Bradbury C., Weeks A. (2001) Parent-based therapies for preschool attention-deficit/hyperactivity disorder: A randomized, controlled trial with a community sample. *Journal of the American Academy of Child and Adolescent Psychiatry*. 40(4):402-8.

Spencer T., Biederman J., Wilens T., Harding M., O’Donnel D., Griffin S. (1996) Pharmacotherapy of attention-deficit hyperactivity disorder across the life cycle. *Journal of the American Academy of Child and Adolescent Psychiatry.* 35(4) 409-32.

Swanson JM., Lerner M., Williams L. (1995) More frequent diagnosis of attention deficit-hyperactivity disorder. *New England Journal of Medicine*. 333:944

Swanson JM., Hinshaw SP., Arnold LE., et al. (2007) Secondary evaluations of MTA 36-month outcomes: propensity score and growth mixture model analysis. *Journal of the American Academy of Child and Adolescent Psychiatry*. 46(8) 1012-1013

Swanson, JM. (2009) Presentation of MTA 10 year outcome. Presented at the ISRCAP conference in Seattle, June 2009.

Tannock R., Martinussen R. (2001) Reconceptualizing ADHD*. Educational Leadership*. 59(3): 20-25

Target M., Fonagy P., Shmueli-Goetz Y. (2003) Attachment representations in school-age children: The development of the child attachment interview (CAI). *Journal of Child Psychotherapy* (2) 171–186.

Tutty S., Gephart H., Wurzbacher K. (2003) Enhancing behavioral and social skill functioning in children newly diagnosed with attention-deficit hyperactivity disorder in a paediatric setting. *Developmental and Behavioral Pediatrics*. 24(1): 51-57.

US Food and Drug administration (2006)

Whalen CK. & Henker B. (1985) The social worlds of hyperactive (ADHD) children. Clinical Psychology Review. 5:447-78.

WHO (1992) The ICD-10 classification of mental and behavioural disorders. Clinical descriptions and diagnostic guidelines. Genéve.

Wolraich ML., McGuinn L., Doffing M. (2007) Treatment of attention deficit hyperactivity disorder in children and adolescents: Safety considerations. *Drug Safety*. 30:17-26.
